# A Product Shape Design and Evaluation Model Based on Morphology Preference and Macroscopic Shape Information

**DOI:** 10.3390/e23060639

**Published:** 2021-05-21

**Authors:** Peng Lu, Shih-Wen Hsiao, Fan Wu

**Affiliations:** Department of Industrial Design, National Cheng Kung University, Tainan 70101, Taiwan; lupeng900917@gmail.com (P.L.); wuxiaofan999@gmail.com (F.W.)

**Keywords:** product design, design methods, aesthetics, evaluation, quadratic curvature entropy

## Abstract

A product form design and evaluation model are proposed. In this method, the forms can be evaluated by three sub-evaluation models which can help designers to grasp consumers’ preferences. In the process, the overall shape is first disassembled into several shape elements, and a morphological chart is constructed. Then, the priority of shape elements is analyzed through the fuzzy analytical hierarchy process, and several required combinations are selected from a morphological chart. Moreover, the fuzzy comprehensive evaluation (I), preference questionnaire (II), and quadratic curvature entropy (III) of the selected combinations are analyzed. Finally, the results of I, II, and III are compared. In conclusion, the three sub-evaluation systems are consistent, which confirms the applicability of the proposed model.

## 1. Introduction

According to the product life cycle theory, different design strategies should be adopted in different development periods to achieve sustainable development. Especially in the maturity stage, the number of competitors will increase, the market competition will become more intense, and the competitive version of the product will reach a high peak. At this time, it is not easy to make a breakthrough in technology, but it is easier to make a breakthrough in shape diversity [1]. At present, the technological diversity of enterprises in manufacturing procedures, product performance, and safety is gradually reduced, but the appearance of products that meet the aesthetic needs of consumers has become a new competitive advantage for enterprises. The appearance, price, and function of a product affect consumers’ purchasing desires and preferences, while ordinary consumers tend to want an attractive appearance [2,3]. Ranscombe et al. [4] pointed out that the appearance of a product will affect its sales and even the market’s lifeblood. Tang et al. [5] believe that appearance and aesthetics are the key points in product design because the shape of the product will intuitively convey its image and affect the consumer’s first impression of the product. Therefore, companies need to design product shapes that meet consumers’ aesthetic preference. Additionally, Forslund and Söderberg [6], in the proposed total product design concept, showed that different shape elements in the overall shape of the product would affect consumers’ purchase intentions. Thus, it is necessary to disassemble the overall shape into multiple shape elements for further analysis. In recent years, many scholars have focused on constructing the relationship between product shape and its image vocabulary [7,8,9,10], and through various techniques [11] to clarify the image demands of consumers for product shape, and then apply it to the shape design and design evaluation of the product. Although this research can satisfy the image demands of consumers for different product shapes, it cannot produce many product shapes. In this study, a morphological database was constructed based on the original and new morphological charts, from which hundreds of combinations of shapes can be formed theoretically, and then the shapes of consumer preference can be selected from them.

In the research of product shape design, scholars mostly use a 2D curve to define the appearance and features. Since 1988, many studies on 2D curves have been accumulated. Biederman and Ju [12] studied 2D curves earlier, proposing that 2D curved profiles have the most significant influence on human perception and recognition of product shape. However, when the vertices of the contour line are reduced, or the middle part of the contour line is removed, the product shape is challenging to identify. Hui and Li [13] proposed a shape blending method based on 2D curves. Subsequently, Hsiao et al. [14] used four different mathematical calculation methods to blend the 2D curves of shape elements from two different products and then to quickly obtain an innovative product form. In the latest research, Hsiao et al. [15] applied this method to yacht design by mixing the shape of Taiwan cultural elements with the shape of a yacht, and finally obtained a yacht shape with the image of Taiwan culture. In addition, based on the concept of shape grammar, many scholars use 2D curves to represent the overall shape of the product and maintain the brand characteristics of the shape through various methods. Pugliese and Cagan [16] described and defined the geometric rules of the shape with the shape grammar of the product to maintain the brand characteristics of the shape. They used the motorcycle as a design case to verify the feasibility of the proposed method. McCormack et al. [17] take Buick as an example to encode its key shape elements into a reusable method, which can be used to maintain consistency between shapes. Cheutet et al. [18] proposed a geometric operation method based on shape grammar, that is, analysis and parameterization of 2D curves, so that car designers can decompose and reconstruct new shapes through the obtained geometric parameters. In similar studies, Hsiao et al. [19] proposed a new method for disassembling and reconstructing 2D curves, which can help designers get a unique shape to maintain the brand characteristics of the automobile. Considering that this paper aims to construct a model for evaluating product shape, 2D curves are still used to define the overall shape and shape elements.

Previous studies have shown that humans tend to perceive the overall shape of objects [20,21,22], so it is necessary to explore the relationship between human perception of product shape and the 2D curve representing the overall shape of the product. However, it is difficult to control the overall shape characteristics of 2D curved profiles using traditional microscopic shape information (e.g., circumference, area, roundness, maximum radius vector, and average radius vector) [23]. Consequently, it depends on the experience and intuition of designers. Fortunately, Ujiie et al. [24] have proposed three types of macroscopic shape information that can be used to evaluate a curved profile, namely, angle entropy, curvature entropy, and quadratic curvature entropy. Meanwhile, they proved that the quadratic curvature entropy is more consistent with human cognition of shape. In this paper, quadratic curvature entropy is used as the criterion to evaluate the overall shape of the product.

This study aims to construct a design and evaluation model of product shape based on the 2D curve and to confirm its effectiveness. An outline of this paper is given as follows. Section 2 describes in detail the research methods and theories involved in this study. Section 3 describes the framework of the research process and the specific implementation steps. Section 4 takes a two-wheel balancing vehicle as the design case and evaluates its shape from three sub-evaluation systems, including the fuzzy comprehensive evaluation system (ES-I), the consumer perceptual evaluation system (ES-II), and the macroscopic shape information evaluation system (ES-III). Section 5 compares the three evaluation results, then examines the effectiveness of the proposed model, and analyzes other factors that may affect the evaluation. Finally, the last section concludes the article.

## 2. Theoretical Background

To establish a practical and effective product shape design and evaluation model, the overall shape of the target product is evaluated from three different perspectives, namely ES-I, ES-II, and ES-III. The research methods and theories involved include morphological analysis, fuzzy analytic hierarchy process, fuzzy comprehensive evaluation, and macroscopic shape information. The details are described below.

### 2.1. Morphological Analysis

Fritz Zwicky [25] proposed the morphological analysis method in 1948, also known as the morphological synthesis method, morphological matrix method, or checkerboard method, which can objectively deal with the details of the problem, more accurately grasp the problem, and make a comprehensive analysis. Theoretically, hundreds of combinations of shapes can be obtained through morphological analysis, so the morphological chart can also be regarded as a solution space [26]. Many scholars use morphological analysis as a technical means to carry out research, covering a wide range of fields, such as design engineering [27], manufacturing engineering [28], industrial engineering [29], and ergonomics [30]. Thus, it is confirmed that morphological analysis is a scientific and practical research method that can be used to analyze all possible combination shapes of a product. Therefore, this article uses morphological analysis to disassemble the overall shape of the target product into several shape elements. The detailed operating procedures are as follows:

1. Disassemble the overall shape of the target product and list the main shape elements;
2. List the achievable element types for each shape element;
3. Construct a morphological chart;
4. Select the option and determine the best combination shape.

In addition, when constructing the morphology chart, the angle at which the shape elements are presented is essential for consumer identification. Chen [31] showed that humans mainly identify objects from their canonical angle, which means the most easily recognizable angle.

### 2.2. Fuzzy Analytical Hierarchy Process (FAHP)

The fuzzy analytic hierarchy process (FAHP) combines fuzzy theory and analytical hierarchy process (AHP) and is good at dealing with fuzzy decision problems [32,33,34]. Saaty [35] proposed AHP in 1980 to deal with decision-making problems with multiple criteria in uncertain environments. AHP determines the weight value of the evaluation criteria by pairwise comparison, which is more rigorous than the earlier method of obtaining the weight value through a simple questionnaire. The operation of AHP can be divided into the following five steps:

1. Define the decision-making issues;
2. Create a hierarchical structure;
3. Create a pairwise comparison matrix (Table 1 shows the measurement and relative importance of AHP);
4. Calculate the eigenvalues;
5. Perform a conformance test.

In the consistency test in step 5, Saaty recommends using the consistency coefficient C.R. for testing. If C.R. is less than 0.1, it means that the pairwise matrix is consistent, and the equation of C.R. is as follows:

(1) $$C.R.=\frac{C.I.}{R.I.},\;C.R.<0.1\Rightarrow \mathrm{OK}$$

R.I. is a random index, the value of which varies with the increase of the standard number, as shown in Table 2. Consistency index C.I. as in Equation (2)

(2) $$C.I.=\frac{\lambda_{max}-n}{n-1}$$

where *λ_max_* is the maximum eigenvalue of the pairwise comparison matrix and *n* is the order of the matrix.

Buckley [36] proposed the FAHP based on the Saaty study, using trapezoidal fuzzy numbers to represent the relative importance of the two elements, and then the fuzzy weights were calculated by the geometric mean method. Mon et al. [37] pointed out that the measurement of traditional AHP is too subjective, so an entropy weight-based FAHP was proposed. The implementation steps of FAHP are similar to conventional AHP, but FAHP needs to be defused and normalized [38]. In this article, the degree of consumer preference for shape is a fuzzy concept. Therefore, a comparative questionnaire is set up by FAHP to obtain the weight value of each shape element of the target product.

### 2.3. Fuzzy Comprehensive Evaluation (FCE)

Because the same thing has many attributes and is influenced by many factors, it is necessary to consider many relevant factors in evaluation. If the fuzzy factor is involved in the evaluation process, it is called fuzzy comprehensive evaluation (FCE) [39]. FCE is good at careful consideration and assessment of many related factors. Several scholars have used FCE to design elderly shopping carts [40], mechanical products [41], and public fitness equipment [42], and have proved that FCE is an objective and effective decision-making method. Because the shape problem is fuzzy, the fuzzy concept can be quantitatively processed by combining FAHP and FCE to facilitate the subsequent comparison and analysis. FCE generally includes the following five steps:

1. Establish the factor set;
2. Establish the weight set;
3. Establish the evaluation set;
4. Build the single factor evaluation matrix;
5. Conduct a fuzzy comprehensive evaluation.

### 2.4. Macroscopic Shape Information

#### 2.4.1. Macroscopic Shape Information of 2D Curves

In information theory, information source consists of a series of source symbols [43,44]. The information content *I_τ_* can be defined by the probability that the source symbol *s_τ_* appears in a series of source symbols *p_τ_*.

(3) $$I_{\tau }=-\log_{2}p_{\tau }\;\;\left(p_{\tau }=P\left[s_{\tau }\right]\right)$$

where *P*[*A*] is the possibility of the occurrence of event *A*.

In addition, the average information content can be used to measure the average uncertainty of information content, which is the same as the equation called entropy used in thermodynamics, defined as:

(4) $$H=\overset{\Lambda }{\underset{\tau =1}{\sum }}p_{\tau }I_{\tau }=-\overset{\Lambda }{\underset{\tau =1}{\sum }}p_{\tau }\log_{2}p_{\tau }$$

where Λ is the number of source symbols. In the present study, the shape curve of the target product is assumed to be an information source, and entropy is the macroscopic shape information. Ujiie et al. [24] used angle entropy, curvature entropy, and quadratic curvature entropy to define the macroscopic shape information, and found that the quadratic curvature entropy is more consistent with human cognition of shape through statistical analysis. Therefore, this paper evaluates the shape curve of a product by quadratic curvature entropy. Specifically, the 2D curved profile of the target product is regarded as the information source, and the value of the quadratic curvature entropy is regarded as its macroscopic shape information.

#### 2.4.2. Definition of the Quadratic Curvature Entropy

The cognitive study of curves shows that humans can be aware of the change of curvature of a curve [45]. If the Markov process is introduced based on the traditional curvature entropy, the change of curvature process can be taken into account, which can improve the human cognitive ability to the macroscopic shape information of the curve. In the series of source symbols, the connection between source symbols is often constrained [43,44]. At this time, the occurrence probability of the source symbol is called the transition probability, which is determined by the previous state, and this random process is called the Markov process. Thus, the information content can be defined as:

(5) $$I_{\tau }=-\log_{2}q_{v,\tau }\;\;\left(q_{v,\tau }=P\left[s_{\tau }|s_{v}\right]\right)$$

where *s_τ_* and *s_v_* are the source symbols and *q_v,τ_* is the translation probability of the source symbol transferred from *s_v_* to *s_τ_*.

**Figure 1:**
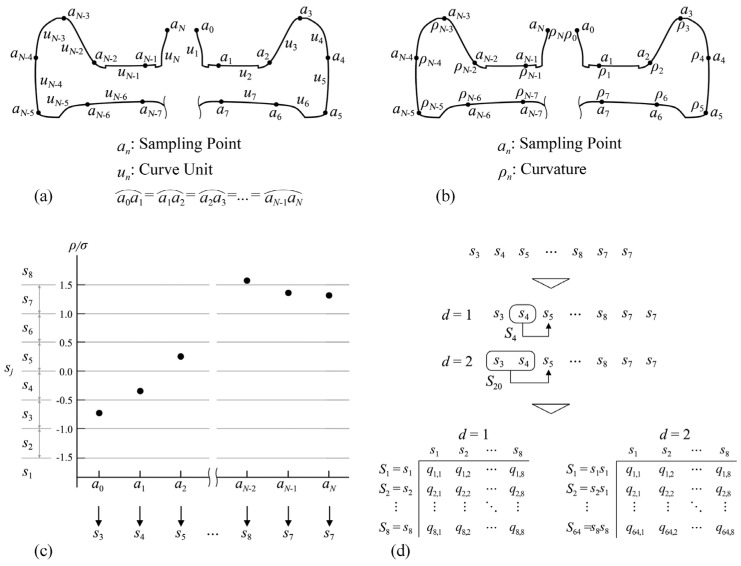
Quadratic curvature entropy of macroscopic shape information: (**a**) sampling point and curve unit; (**b**) sampling of the curvature; (**c**) quantization based on the curvature; (**d**) calculation of the transition probability.

Thus, the entropy based on the Markov process is defined as:

(6) $$H_{m}=\overset{\Lambda^{\delta }}{\underset{v=1}{\sum }}\overset{\Lambda }{\underset{\tau =1}{\sum }}q_{v}q_{v,\tau }I_{v,\tau }=-\overset{\Lambda^{\delta }}{\underset{v=1}{\sum }}\overset{\Lambda }{\underset{\tau =1}{\sum }}q_{v}q_{v,\tau }\log_{2}q_{v,\tau }$$

where *q_v_* is the probability of the occurrence of a state, *δ* is the number of source symbols within a state, and Λ is the number of source symbol types. In this paper, the shape curve of the product is assumed to be the information source so that the macroscopic shape information could be calculated. Firstly, the curved profile is divided into *N* equivalent curve units by sampling points (Figure 1a), and the curvature *ρ_n_* in each sampling point is calculated (Figure 1b). Secondly, a set of source symbols is constructed from the value of (*ρ_n_*/*σ*), where *σ* is the standard deviation of curvature. Thirdly, the range of the value (*ρ_n_*/*σ*) is set from −1.5 to 1.5, and the number of source symbols *V* is 8 (Figure 1c). Fourthly, calculate *q_i_* (the occurrence probability of state *Si*) and *q_i,j_* (the transition probability of source symbol *s_j_* in state *S_i_*) (Figure 1d). *d* denotes the number of source symbols that form a state, and in this paper, it is set to 1. According to the information theory, the parameters mentioned above can be used to calculate the quadratic curvature entropy *H_QC_* of the curve; the formula is as follows:

(7) $$H_{QC}=-\frac{1}{\log_{2}V}\overset{V^{d}}{\underset{i=1}{\sum }}\overset{V}{\underset{j=1}{\sum }}q_{i}q_{i,j}\log_{2}q_{i,j}\;\;\left(0\leq H_{QC}\leq 1\right)$$

To ensure that the variation range is between 0 and 1, *H_QC_* is divided by the maximum entropy log_2_*V*. The value of the quadratic curvature entropy of the overall shape curve can be calculated through the above four steps.

## 3. Implementation Procedures

**Figure 2:**
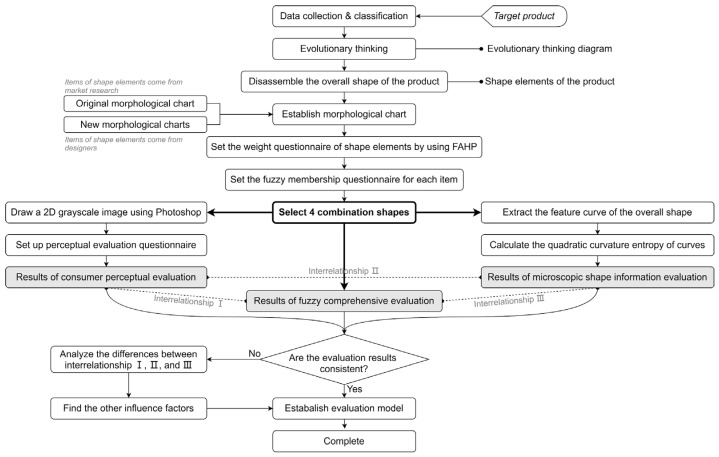
The framework of the shape evaluation model.

Based on the theory described in Section 2, this study aimed to construct a design and evaluation model of product shape, using the two-wheel balancing vehicle as a case study. The research flow framework is shown in Figure 2, and the specific implementation procedures are described below.

1. Identify target products, collect information and classify them;
2. Collect products related to the target product and create an evolutionary thinking diagram;
3. Decompose the overall shape of the target product into a limited number of shape elements;
4. Create a morphological chart with original and newly designed shape types;
5. Set up a questionnaire to clarify the importance of each shape element;
6. Set up a questionnaire to clarify the fuzzy membership of each type in the shape elements;
7. According to the maximum and minimum membership principle, select four combinations from the established morphological chart;
8. Conduct a fuzzy comprehensive evaluation of four combinations;
9. Conduct consumer perception evaluation of four combinations;
10. Conduct macroscopic shape information evaluation of four combinations, and the quadratic curvature entropy of the overall shape curve is used as the evaluation index.

## 4. Case Study

The complexity of the two-wheel balancing vehicle is moderate, so it is generally represented as a case of this study. This paper evaluates the two-wheel balancing vehicle from three different perspectives, and the validity of the proposed evaluation model is verified by pairwise comparison. The specific implementation steps are described in Section 3.

### 4.1. Description and Evolutionary Thinking of the Target Product

**Figure 3:**
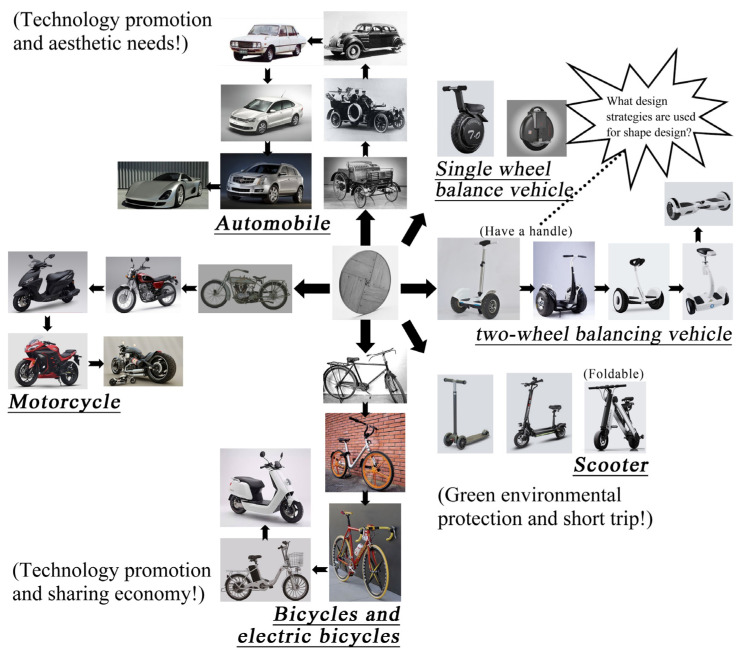
The evolutionary thinking diagram for the target product.

The first balancing vehicle came from the U.S. Segway. After years of development, the production of two-wheel balancing vehicles has grown year by year, resulting in increasingly fierce competition among brands [46]. From the current development situation, it can be inferred that the two-wheel balancing vehicle is a mature product, so its design needs to consider the diversity of shape to extend its life cycle, thereby bringing more benefits to the enterprise. To have a more comprehensive understanding of the history and evolution of the target product and to predict its future direction, the “wheel” was used as the origin to draw an evolutionary thinking diagram (Figure 3) of seven products (motorcycle, bicycle, automobile, single-wheel balancing vehicle, two-wheel balancing vehicle, electric car, and electric scooter) of the same family. By contemplating the diagram, we could propose what kind of design strategy should be developed for a two-wheel balancing vehicle. In addition, considering that the two-wheel balancing vehicle is in the mature stage, the shape of consumer preference should be taken as the breakthrough point of the design. In other words, it is crucial to evaluate the shape of the product accurately at the design stage.

### 4.2. Constructing a Morphological Chart for the Two-Wheel Balancing Vehicle

In this phase, the original and the new morphological charts are established based on the existing and newly designed shape elements. This study focuses on the overall shape of the product, and other factors that affect consumer preferences, such as color, decals, and decorations, are beyond the scope of this study. Therefore, the shape elements of the target product were drawn with 2D curves, and the front view was selected as its canonical view and presented in the form of a grayscale diagram. First, the overall shape of the two-wheel balancing vehicle was disassembled into a combination of five shape elements, namely, handle, steering rod, body, pedal, and wheel, which can be expressed as *U* = {handlebar, steering rod, body, pedal, wheel}. Then, we combined the product images collected in the previous market research to construct the original morphological chart, as shown in Table 3. In addition, based on recognizing the shape of two-wheel balancing vehicles, the design team designed a series of new types for the shape elements based on their aesthetic intuition and previous design experience. A new morphological chart was constructed with new types after focus group discussions, as shown in Table 4. Finally, we merged the two morphological charts, as shown in Table 5.

### 4.3. Clarifying the Weight of Each Shape Element

Different shape elements in the overall shape affect consumers’ purchase intention [14], and the preference degree of shape elements belongs to the fuzzy concept. Thus, this part sets the preference questionnaire with FAHP to obtain the relative weight value of each shape element. Finally, 90 valid questionnaires were collected, and the subjects aged 18–35 participated in the survey, including product design students, designers, and general balance vehicle users. The ratio of males to females was 6:4. A pairwise comparison matrix determines the weight values of all shape elements, and the calculated results are shown in Table 6. The geometric average of the row vector method was adopted, and the normalized value was used as the final weight value, as shown on the right side of Table 6. 

To ensure that the weight values of each shape element are consistent, a consistency test is required. In the pairwise comparison matrix in Table 6, the sum of the correlation coefficient multiplied by the weight of each shape element is the *λ* value of each shape element, and the largest eigenvalue *λ*_max_ of this matrix is calculated as follows:

$$\lambda_{max}=\left(\frac{5.178+5.107+5.122+5.132+5.213}{5}\right)=5.150$$

We incorporate *λ*_max_ into Equations (1) and (2) to calculate C.R.

$$C.I.=\frac{\lambda_{max}-n}{n-1}=\frac{5.150-5}{4}=0.0375$$

$$C.R.=\frac{C.I.}{R.I.}=\frac{0.0375}{1.12}=0.033<0.1\Rightarrow \mathrm{OK}$$

The calculation results show that the weight values of each shape element are consistent so the weight set can be expressed as:

$$A=\frac{a_{1}}{u_{1}}+\frac{a_{2}}{u_{2}}+\frac{a_{3}}{u_{3}}+\frac{a_{4}}{u_{4}}+\frac{a_{5}}{u_{5}}=\frac{0.286}{\mathrm{Handlebar}}+\frac{0.286}{\mathrm{Steering}\;\mathrm{rod}}+\frac{0.286}{\mathrm{Body}}+\frac{0.286}{\mathrm{Pedals}}+\frac{0.286}{\mathrm{Wheel}}\;=\left(0.286,\;0.150,\;0.196,\;0.152,\;0.216\right)$$

From the above results, it can be seen that the importance attached by subjects to the evaluation shape elements is represented as handle > wheel > body > pedal > steering rod.

### 4.4. Identifying the Fuzzy Membership of Each Type of Shape Element

Taking the set of shape elements in Section 4.2 as the factor set, it can be expressed as *U* = {handlebar (*u*_1_), steering rod (*u*_2_), body (*u*_3_), pedals (*u*_4_), wheel (*u*_5_)}. Then, according to the factor set and weight set, a comprehensive assessment questionnaire was set to obtain the fuzzy membership of each type. A total of 124 subjects aged 18–35 participated in the survey, including general balancing vehicle users, teachers and students majoring in product design, and consumers willing to buy the balancing vehicle. The ratio of males to females was 5:5. Among all subjects, more than 80% were general balancing vehicle users and consumers willing to buy the balancing vehicle. After statistical analysis, the fuzzy membership of each type is shown in Table 7.

### 4.5. Four Combinations Are Selected from the Merged Morphological Chart

**Figure 4:**
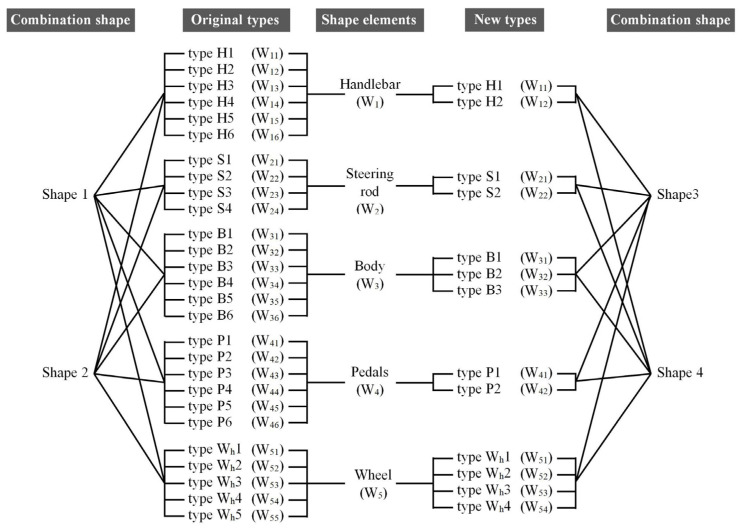
The hierarchical structure of four combination shapes.

**Figure 5:**
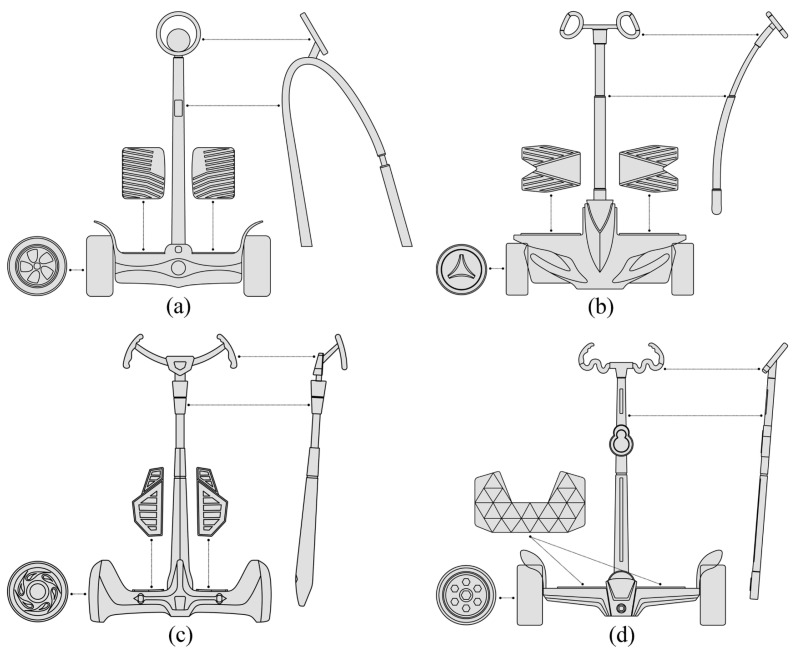
Assembly effect diagram: (**a**) shape 1; (**b**) shape 2; (**c**) shape 3; (**d**) shape 4.

Two combinations were selected from the original morphological chart and the new morphological chart, respectively. A total of four combinations were used as the evaluation samples, and the hierarchical structure of the four combinations can be expressed as shown in Figure 4. The specific selection criteria were to select the types with the most significant and smallest fuzzy memberships from the two morphological charts, respectively. When the membership values are the same, one of them is randomly selected, so the four combinations can be expressed as shown in Table 8 and Table 9, and the corresponding assembly effect is shown in Figure 5. Four different combinations of the two-wheel balancing vehicle are:

Shape 1: Assembled from type H6, type S2, type B5, type P3, and type W_h_4 of the height fuzzy memberships (Figure 5a).
Shape 2: Assembled from type H2, type S4, type B1, type P1, and type W_h_2 of the lowest fuzzy memberships (Figure 5b).
Shape 3: Assembled from type H1, type S1, type B2, type P2, and type W_h_3 of the height fuzzy memberships (Figure 5c).
Shape 4: Assembled from type H2, type S2, type B3, type P1, and type W_h_1 of the lowest fuzzy memberships (Figure 5d).

### 4.6. Establish a Variety of Evaluation Systems

To ensure that the evaluation results are the same as the true feelings of consumers, this stage was evaluated from three different perspectives, namely, fuzzy comprehensive evaluation (ES-I), consumer perceptual evaluation (ES-II), and macroscopic shape information evaluation (ES-III).

#### 4.6.1. ES-I: The Fuzzy Comprehensive Evaluation System

According to Section 2.3, the factor set can be expressed as *U* = (handlebar (*u*_1_), steering rod (*u*_2_), body (*u*_3_), pedals (*u*_4_), wheel (*u*_5_)), and the weight set can be expressed as *A* = (0.286, 0.150, 0.196, 0.152, 0.216). From Table 7, the fuzzy membership of each type of the four combinations can be represented as shown in Table 10. To facilitate the operation of the fuzzy comprehensive evaluation, the fuzzy membership of each type can be further transformed into a fuzzy evaluation matrix, that is, the single factor evaluation set can be expressed as

$$\begin{array}{c} R_{1}=\left[0.145\;0.226\;0.089\;0.129\;0.194\right]\\ R_{2}=\left[0.089\;0.056\;0.008\;0.048\;0.056\right]\\ R_{3}=[0.274\;0.234\;0.500\;0.298\;0.105]\\ R_{4}=[0.064\;0.185\;0.121\;0.161\;0.048] \end{array}$$

Finally, the fuzzy evaluation matrix set R can be expressed as

$$R=\left[\begin{array}{cc} \begin{array}{c} 0.145\\ \begin{array}{c} 0.226\\ \begin{array}{c} 0.086\\ \begin{array}{c} 0.129\\ 0.194 \end{array} \end{array} \end{array} \end{array} & \begin{array}{cc} \begin{array}{c} 0.089\\ 0.056\\ \begin{array}{c} 0.008\\ \begin{array}{c} 0.048\\ 0.056 \end{array} \end{array} \end{array} & \begin{array}{cc} \begin{array}{c} 0.274\\ \begin{array}{c} 0.234\\ \begin{array}{c} 0.500\\ \begin{array}{c} 0.298\\ 0.105 \end{array} \end{array} \end{array} \end{array} & \begin{array}{c} 0.064\\ 0.185\\ \begin{array}{c} 0.121\\ \begin{array}{c} 0.161\\ 0.048 \end{array} \end{array} \end{array} \end{array} \end{array} \end{array}\right]$$

The single factor fuzzy evaluation only reflects the influence of one factor on the evaluation object. If the effect of all factors can be considered comprehensively, the correct evaluation result can be obtained so that the fuzzy comprehensive evaluation set can be expressed as Formula (8):

(8) $$B=A\circ R=\left[b_{1},b_{2},\ldots b_{j},\ldots b_{m}\right]$$

where the symbol “∘” represents the fuzzy composition operation, *A* is the weighted set of shape elements, and *b* is called the fuzzy comprehensive evaluation index. The fuzzy composition method *M*(˄,˅) is used in this article [47], as shown in Formula (9).

(9) $$b_{j}=\overset{m}{\underset{i=1}{\vee }}\left(a_{i}\wedge r_{ij}\right);j=1,2,\ldots ,n$$

where “˅” and “˄” represent big-choosing and small-choosing, respectively.

Therefore, the fuzzy comprehensive evaluation set of the four combinations can be represented as:

$$B=A\circ R=\left[\left(0.286,\;0.150,\;0.196,\;0.152,\;0.216\right)\right]\circ \left[\begin{array}{ccc} \begin{array}{c} 0.145\\ 0.226\\ \begin{array}{c} 0.089\\ 0.129\\ 0.194 \end{array} \end{array} & \begin{array}{c} 0.089\\ 0.056\\ \begin{array}{c} 0.008\\ 0.048\\ 0.056 \end{array} \end{array} & \begin{array}{cc} \begin{array}{c} 0.274\\ 0.234\\ \begin{array}{c} 0.500\\ 0.298\\ 0.105 \end{array} \end{array} & \begin{array}{c} 0.064\\ 0.185\\ \begin{array}{c} 0.121\\ 0.161\\ 0.048 \end{array} \end{array} \end{array} \end{array}\right]\;=\left(0.154\;0.055\;0.279\;0.105\right)$$

After normalizing, the above vector is (0.260, 0.093, 0.470, 0.177). From the above results, it can be seen that the priority order of the four combinations is shape 3, shape 1, shape 4, and shape 2.

#### 4.6.2. ES-II: Consumer Perceptual Evaluation System

In this stage, four combinations were used as the sample to set up a consumer perceptual questionnaire. A total of 100 subjects aged 18–30 participated in the survey, mainly composed of general balancing vehicle users and consumers willing to buy the balancing vehicle. The ratio of males to females was 5:5. During the questionnaire survey, the subjects were required to score the four combinations from high to low according to their personal preferences, and the score range was between 0 and 1. After statistical analysis, the result is shown in Table 11. The results show that the order between the four combinations is as follows: shape 3 > shape 1 > shape 4 > shape 2.

#### 4.6.3. ES-III: Macroscopic Shape Information Evaluation System

Section 2.4 has shown that the quadratic curvature entropy is regarded as the macroscopic shape information of a curved profile, which can evaluate the human macroscopic perception of the curved profile. At this stage, the overall shape contour curve of the target product is regarded as the information source in information theory. In addition, considering that the cross points and nondifferentiable points on the contour could affect the evaluation, a cubic Bézier curve [48,49] with a continuous and differentiable connection point was used to plot the overall contour. The specific method is to calculate the quadratic curvature entropy of the curved profiles of the four combinations, respectively. The detailed operating procedures are described below.

**Figure 6:**
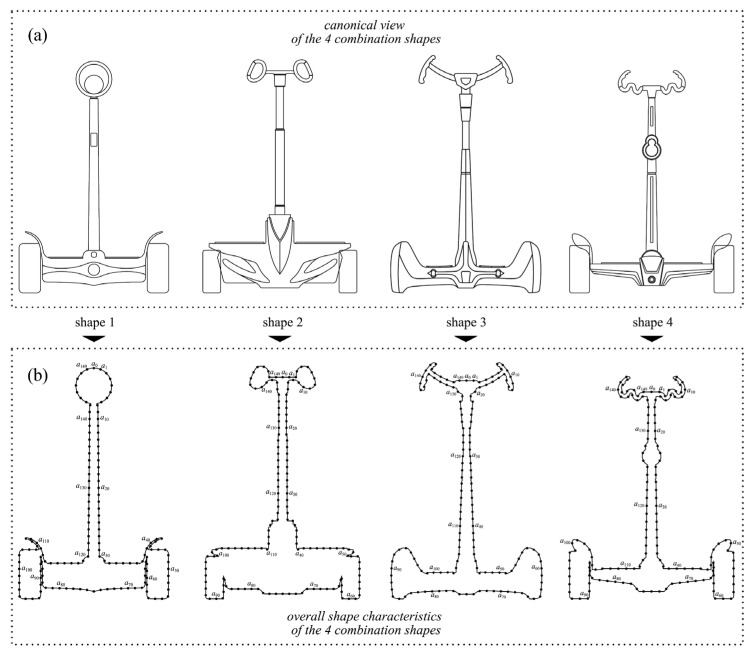
Extracting the overall shape characteristics of the canonical view: (**a**) canonical view of the four shapes; (**b**) overall shape characteristics and its sampling points.

1. Using the cubic Bézier curve to plot the overall contours of the four combinations based on the assembly effect diagrams (Figure 6a);
2. Using 150 sampling points to divide each closed curved profile into 150 equal curve units (Figure 6b) and calculating the curvature *ρ_n_* at the location of each sampling point;
3. The source symbols set of the four curve contours are constructed with the value of (*ρ_n_*/*σ*), and the number of source symbols *V* is set to 8. In addition, this study only considered the effect of a previous source symbol, so the value of *d* was set to 1;
4. After calculation, the occurrence probabilities *q_i_* of source symbols of the four curve contours are shown in Table 12, and the transfer probabilities *q_i,j_* are shown in Table 13, Table 14, Table 15 and Table 16, and then the data were brought into Formula (7) to obtain the values of the quadratic curvature entropy of the four curve contours. The calculation results are shown below.

$$\begin{array}{c} H_{QC1}=-\frac{1}{\log_{2}V}\sum_{i=1}^{V^{d}}\sum_{j=1}^{V}q_{i}q_{i,j}\log_{2}q_{i,j}=-\frac{1}{\log_{2}8}\sum_{i=1}^{8^{1}}\sum_{j=1}^{8}q_{i}q_{i,j}\log_{2}q_{i,j}=0.309\\ H_{QC4}=-\frac{1}{\log_{2}V}\sum_{i=1}^{V^{d}}\sum_{j=1}^{V}q_{i}q_{i,j}\log_{2}q_{i,j}=-\frac{1}{\log_{2}8}\sum_{i=1}^{8^{1}}\sum_{j=1}^{8}q_{i}q_{i,j}\log_{2}q_{i,j}=0.345\\ H_{QC1}=-\frac{1}{\log_{2}V}\sum_{i=1}^{V^{d}}\sum_{j=1}^{V}q_{i}q_{i,j}\log_{2}q_{i,j}=-\frac{1}{\log_{2}8}\sum_{i=1}^{8^{1}}\sum_{j=1}^{8}q_{i}q_{i,j}\log_{2}q_{i,j}=0.324\\ H_{QC1}=-\frac{1}{\log_{2}V}\sum_{i=1}^{V^{d}}\sum_{j=1}^{V}q_{i}q_{i,j}\log_{2}q_{i,j}=-\frac{1}{\log_{2}8}\sum_{i=1}^{8^{1}}\sum_{j=1}^{8}q_{i}q_{i,j}\log_{2}q_{i,j}=0.341 \end{array}$$

A smaller entropy value indicates that the curvature change of the contour curve is more stable, and it also shows that the contour curve is more in line with human aesthetic preferences. Therefore, the priority order of the above four combinations is shape 1, shape 3, shape 4, and shape 2.

## 5. Results and Discussion

**Figure 7:**
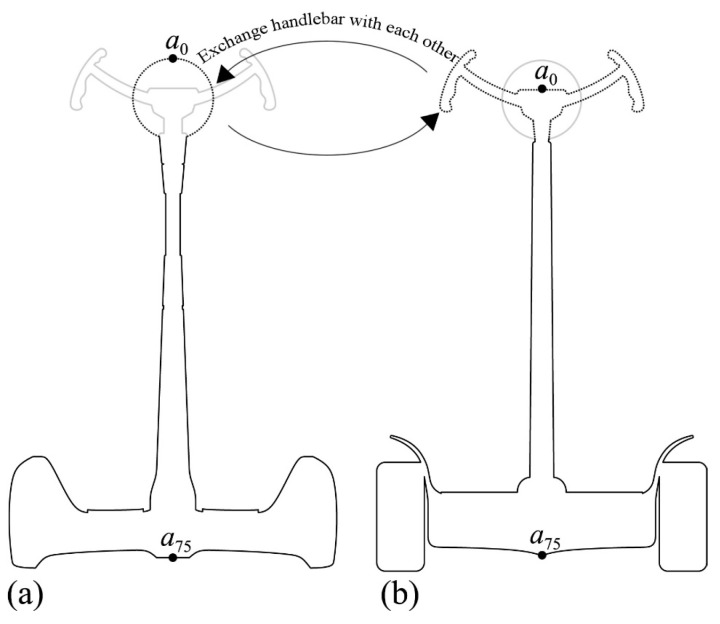
Two shapes exchange handlebars with each other: (**a**) shape 3 with the handlebar of shape 1; (**b**) shape 1 with the handlebar of shape 3.

From Section 4.6, it can be seen that the results of the three evaluations are highly consistent, in which the result of the fuzzy comprehensive evaluation is entirely consistent with the result of consumer perceptual evaluation, all of which are shape 3 > shape 1 > shape 4 > shape 2, indicating that the degree of consumer preference for each combination can be accurately evaluated based on those relative weights of shape elements (Table 6) and those fuzzy memberships of their types (Table 7). In other words, the order of preferences for hundreds of combinations can be obtained according to Table 6 and Table 7. However, the evaluation result of macroscopic shape information is slightly different from the first two, which is reflected in the reverse order of shape 1 and shape 3. Therefore, it is still necessary to analyze the causes of the errors and further explore the potential influencing factors. As can be seen from Figure 6b, there is a clear difference between the handlebar of shape 1 and the handlebars of the other three shapes, which is embodied in the fact that the handlebar of Shape 1 is relatively simple (i.e., the curvature change is small), and the handlebar of shape 3 is more complicated (i.e., the curvature changes significantly), thus assuming that this is the cause of the error. To verify the correctness of the hypothesis, the handlebars of the two shapes were exchanged (Figure 7), and then the macroscopic shape information of the two curve contours after the exchange was calculated. The calculation results show that the entropy value of the new shape 3 (i.e., with the handlebar of shape 1) is smaller than the entropy value of the new shape 1 (i.e., with the handlebar of shape 3), indicating that the curvature change of the new shape 3 is more stable and more consistent with the aesthetic preference of consumers. Thus, it can be seen that the difference between the element type and other element types leads to the error of macroscopic shape information evaluation and the other two evaluations. Therefore, if there are apparent differences between the types of shape elements in the evaluation process, it can be verified through the above method to obtain a more accurate evaluation result. Furthermore, the canonical angle of the product (Figure 6a) contains more shape information than the overall shape profile of the product (Figure 6b). Although there is no significant effect of this factor in this study, this factor cannot be wholly ignored in the evaluation process of other products.

## 6. Conclusions

This article proposes a product shape design and evaluation model based on the 2D curve of product shape. This model included three sub-evaluation systems: the fuzzy comprehensive evaluation system (ES-I), the consumer perceptual evaluation system (ES-II), and the macroscopic shape information evaluation system (ES-III). ES-I integrated morphological analysis, fuzzy hierarchical analysis (FAHP), and fuzzy comprehensive evaluation (FCE). ES-II conducted a questionnaire survey of users and potential users who use the target product. ES-III combined information theory, the Markov process, and quadratic curvature entropy. It was found that the evaluation results of ES-I and ES-II were entirely consistent, but there were some differences between ES-III and the first two, which was due to the significant difference between the handlebars of the shape elements of shape 1 and the other three shapes. In ES-I, FAHP could clarify the relative importance of each shape element (Table 6), and subsequent questionnaires could clarify the fuzzy membership of each type of shape element (Table 7), while the final fuzzy comprehensive evaluation could help designers quantify and make judgments about perceptual shape preferences. In ES-III, the quadratic curvature entropy closest to human cognition was selected as an index to evaluate the product shape, which could help design engineers without systematic aesthetic training to determine the best product shape quickly. In summary, the three sub-evaluation systems belong to a parallel relationship. If the three evaluation results could be used as a reference in the design process of shape, it could help designers more accurately grasp consumers’ actual shape preferences, thereby helping enterprises reduce design cost and increase product market share.

Humans identify objects based on a preset angle (e.g., the canonical angle of the object) stored in the brain [31]. Because the front view of the two-wheel balancing vehicle contained more shape information, it was taken as the canonical angle to build the morphological chart in this paper. We built a new morphological chart with the newly designed types of shape elements based on the original morphological chart. Finally, we obtained a merged morphological chart. In terms of the morphological chart, the merged morphological chart dramatically increases the total number of combinations of shapes and improves the possibility of shape innovation. Subsequently, it can be seen from the three evaluation results that the combinations from the new morphological chart are better than those from the original morphological chart, except for shape 3 and shape 1 in ES-III, but the reason has been analyzed.

The present study is subject to some limitations. First, since the current product upgrades are very fast, this might cause the proposed shape evaluation model to fall behind the current products on the market. Therefore, it is necessary to promptly update those types of shape elements in the morphological chart to improve the usability of the evaluation model. Second, considering that consumers’ aesthetic demands are constantly changing over time, it is necessary to regularly investigate the users’ preference for each type of shape element in the morphological chart to conduct an accurate and effective evaluation. Lastly, due to the limitation of research conditions, we only used the 2D curve of the product as the evaluation sample. In the subsequent research, if a three-dimensional morphological chart is used instead of the two-dimensional morphological chart, we could get more intuitive combinations of shapes and make a more accurate evaluation. For instance, the macroscopic shape information could be evaluated from different perspectives of the three-dimensional shape combination. Although the two-wheel balancing vehicle was used as a case study in this paper, the proposed design and evaluation model is also suitable for other products.

## Figures and Tables

**Table 1:** Evaluation measurement and relative definition of the analytic hierarchy process.

Evaluation Measurement	Definition
1	Equal importance
3	Slight importance
5	Essential importance
7	Very strong importance
9	Absolute importance
2,4,6,8	Intermediate values

**Table 2:** Table of random indexes.

Order N	3	4	5	6	7	8	9	10
R.I.	0.58	0.9	1.12	1.24	1.32	1.44	1.45	1.49

**Table 3:** The original morphological chart.

Order N	Type 1	Type 2	Type 3	Type 4	Type 5	Type 6
Handlebar	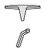	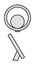	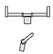	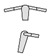	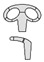	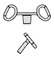
Steering rod	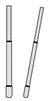	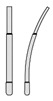	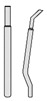	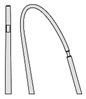		
Body	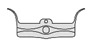	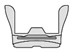	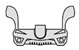	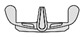	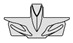	
Pedals	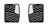	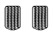	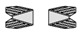	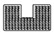	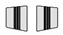	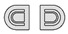
Wheel	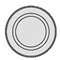	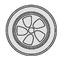	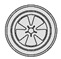	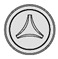	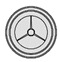	

**Table 4:** The new morphological chart.

Order N	Type 1	Type 2	Type 3	Type 4
Handlebar	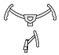	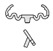		
Steering rod	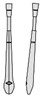	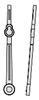		
Body	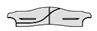	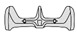	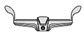	
Pedals	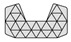	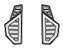		
Wheel	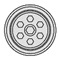	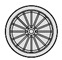	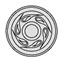	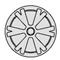

**Table 5:** The merged morphological chart.

	Type 1	Type 2	Type 3	Type 4	Type 5	Type 6	Type 7	Type 8	Type 9
Handlebar	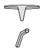	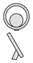	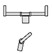	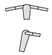	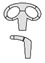	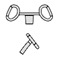	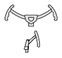	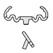	
Steering rod	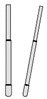	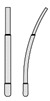	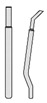	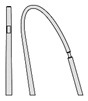	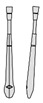	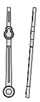			
Body	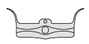	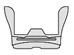	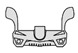	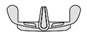	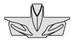	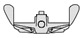	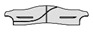	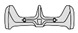	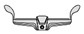
Pedals	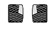	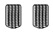	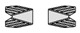	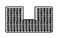	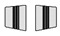	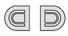	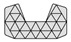	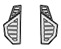	
Wheel	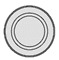	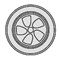	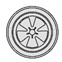	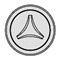	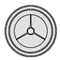	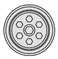	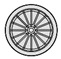	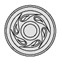	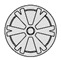

**Table 6:** The pairwise comparison matrix.

	Handlebar (H)	Steering Rod (S)	Body (B)	Pedals (P)	Wheel (W_h_)	Geometric Mean	Weight (W)
Handlebar (H)	1	2.267	1.867	2.111	0.778	1.474	0.286
Steering rod (S)	0.441	1	0.90	0.733	0.937	0.771	0.150
Body (B)	0.536	1.111	1	1.822	0.978	1.012	0.196
Pedals (P)	0.474	1.364	0.549	1	0.833	0.784	0.152
Wheel (W_h_)	1.285	1.067	1.023	1.2	1	1.110	0.216

**Table 7:** The fuzzy membership of each type.

	Type 1	Type 2	Type 3	Type 4	Type 5	Type 6	Type 7	Type 8	Type 9
Handlebar (H)	0.113	0.089	0.113	0.105	0.097	0.145 *	0.274 *	0.064	
Steering rod (S)	0.089	0.226 *	0.21	0.056	0.234 *	0.185			
Body (B)	0.008	0.016	0.032	0.057	0.089 *	0.032	0.145	0.500 *	0.121
Pedals (P)	0.048	0.113	0.129 *	0.065	0.073	0.113	0.161	0.298 *	
Wheel (W_h_)	0.121	0.056	0.121	0.194 *	0.161	0.048	0.097	0.105 *	0.097

On the right side of the dotted line is the fuzzy membership of the new designed types; “*” represents the most significant type of fuzzy membership in the original morphological chart (Table 3) and the new morphological chart (Table 4).

**Table 8:** Combinations with the maximum and minimum fuzzy membership in the original morphological chart.

	Type 1	Type 2	Type 3	Type 4	Type 5	Type 6
Handlebar(H)	H1	H2^min^	H3	H4	H5	H6^max^
Steering rod(S)	S1	S2^max^	S3	S4^min^		
Body(B)	B1^min^	B2	B3	B4	B5^max^	B6
Pedals(P)	P1^min^	P2	P3^max^	P4	P5	P6
Wheel(W_h_)	W_h_1	W_h_2^min^	W_h_3	W_h_4^max^	W_h_5	W_h_6

“max” represents the largest fuzzy memberships; “min” represents the lowest fuzzy memberships.

**Table 9:** Combinations with the maximum and minimum fuzzy membership weight in the new morphological chart.

Order N	Type 1	Type 2	Type 3	Type 4
Handlebar(H)	H1^max^	H2^min^		
Steering rod(S)	S1^max^	S2^min^		
Body(B)	B1	B2^max^	B3^min^	
Pedals(P)	P1^min^	P2^max^		
Wheel(W_h_)	W_h_1^min^	W_h_2	W_h_3^max^	W_h_4

“max” represents the largest fuzzy memberships; “min” represents the lowest fuzzy memberships.

**Table 10:** Fuzzy memberships of the four combination shapes.

Shape Elements	Combination Shape 1	Combination Shape 1	Combination Shape 1	Combination Shape 1
Handlebar (H)	0.145	0.089	0.274	0.064
Steering rod (S)	0.226	0.056	0.234	0.185
Body (B)	0.089	0.008	0.500	0.121
Pedals (P)	0.129	0.048	0.298	0.161
Wheel (W_h_)	0.194	0.056	0.105	0.048

**Table 11:** Statistical score and order table.

	Idea 1	Idea 2	Idea 3	Idea 4
Total score of all questionnaires	58.9	48.4	69.7	57.2
Ranking	2	4	1	3

**Table 12:** Statistical table of the probability of occurrence of a source symbol.

	Shape 1		Shape 2		Shape 3		Shape 4	
Source Symbols	Number	Probability	Number	Probability	Number	Probability	Number	Probability
*s*_1_	4	0.027	2	0.0133	6	0.0400	2	0.0133
*s*_2_	0	0.000	0	0.0000	0	0.0000	2	0.0133
*s*_3_	4	0.027	4	0.0267	10	0.0667	8	0.0533
*s*_4_	59	0.393	82	0.5467	43	0.2867	59	0.3933
*s*_5_	71	0.473	45	0.3000	73	0.4867	63	0.4200
*s*_6_	4	0.027	7	0.0467	12	0.0800	10	0.0667
*s*_7_	4	0.027	8	0.0533	2	0.0133	4	0.0267
*s*_8_	4	0.027	2	0.0133	4	0.0267	2	0.0133

**Table 13:** Transition probability of shape 1.

	*s*_1_	*s*_2_	*s*_3_	*s*_4_	*s*_5_	*s*_6_	*s*_7_	*s*_8_
*s*_1_	Non	Non	Non	*q*_1,4_ = 0.020	Non	Non	*q*_1,7_ = 0.007	Non
*s*_2_	Non	Non	Non	Non	Non	Non	Non	Non
*s*_3_	Non	Non	Non	*q*_3,4_ = 0.013	*q*_3,5_ = 0.013	Non	Non	Non
*s*_4_	*q*_4,1_ = 0.020	Non	*q*_4,3_ = 0.013	*q*_4,4_ = 0.220	*q*_4,5_ = 0.107	*q*_4,6_ = 0.013	*q*_4,7_ = 0.007	*q*_4,8_ = 0.013
*s*_5_	Non	Non	*q*_5,3_ = 0.013	*q*_5,4_ = 0.107	*q*_5,5_ = 0.313	*q*_5,6_ = 0.013	*q*_5,7_ = 0.013	*q*_5,8_ = 0.013
*s*_6_	Non	Non	Non	*q*_6,4_ = 0.013	*q*_6,5_ = 0.013	Non	Non	Non
*s*_7_	*q*_7,1_ = 0.007	Non	Non	*q*_7,4_ = 0.007	*q*_7,5_ = 0.013	Non	Non	Non
*s*_8_	Non	Non	Non	*q*_8,4_ = 0.013	*q*_8,5_ = 0.013	Non	Non	Non

**Table 14:** Transition probability of shape 2.

	*s*_1_	*s*_2_	*s*_3_	*s*_4_	*s*_5_	*s*_6_	*s*_7_	*s*_8_
*s*_1_	Non	Non	Non	*q*_1,4_ = 0.007	Non	Non	*q*_1,7_ = 0.007	Non
*s*_2_	Non	Non	Non	Non	Non	Non	Non	Non
*s*_3_	Non	Non	Non	*q*_3,4_ = 0.013	Non	*q*_3,6_ = 0.013	Non	Non
*s*_4_	*q*_4,1_ = 0.007	Non	*q*_4,3_ = 0.013	*q*_4,4_ = 0.440	*q*_4,5_ = 0.053	*q*_4,6_ = 0.007	*q*_4,7_ = 0.013	*q*_4,8_ = 0.013
*s*_5_	Non	Non	Non	*q*_5,4_ = 0.053	*q*_5,5_ = 0.187	*q*_5,6_ = 0.027	*q*_5,7_ = 0.033	Non
*s*_6_	Non	Non	*q*_6,3_ = 0.013	*q*_6,4_ = 0.007	*q*_6,5_ = 0.027	Non	Non	Non
*s*_7_	*q*_7,1_ = 0.007	Non	Non	*q*_7,4_ = 0.013	*q*_7,5_ = 0.033	Non	Non	Non
*s*_8_	Non	Non	Non	*q*_8,4_ = 0.013	Non	Non	Non	Non

**Table 15:** Transition probability of shape 3.

	*s*_1_	*s*_2_	*s*_3_	*s*_4_	*s*_5_	*s*_6_	*s*_7_	*s*_8_
*s*_1_	Non	Non	*q*_1,3_ = 0.007	*q*_1,4_ = 0.013	*q*_1,5_ = 0.013	*q*_1,6_ = 0.007	Non	Non
*s*_2_	Non	Non	Non	Non	Non	Non	Non	Non
*s*_3_	*q*_3,1_ = 0.007	Non	*q*_3,3_ = 0.027	*q*_3,4_ = 0.027	*q*_3,5_ = 0.020	*q*_3,6_ = 0.007	*q*_3,7_ = 0.007	Non
*s*_4_	*q*_4,1_ = 0.013	Non	Non	*q*_4,4_ = 0.120	*q*_4,5_ = 0.100	*q*_4,6_ = 0.020	Non	*q*_4,8_ = 0.007
*s*_5_	*q*_5,1_ = 0.013	Non	*q*_5,3_ = 0.020	*q*_5,4_ = 0.100	*q*_5,5_ = 0.307	*q*_5,6_ = 0.033	Non	*q*_5,8_ = 0.013
*s*_6_	*q*_6,1_ = 0.007	Non	*q*_6,3_ = 0.007	*q*_6,4_ = 0.020	*q*_6,5_ = 0.033	Non	*q*_6,7_ = 0.007	*q*_6,8_ = 0.007
*s*_7_	Non	Non	*q*_7,3_ = 0.007	Non	Non	*q*_7,6_ = 0.007	Non	Non
*s*_8_	Non	Non	Non	*q*_8,4_ = 0.007	*q*_8,5_ = 0.013	*q*_8,6_ = 0.007	Non	Non

**Table 16:** Transition probability of shape 4.

	*s*_1_	*s*_2_	*s*_3_	*s*_4_	*s*_5_	*s*_6_	*s*_7_	*s*_8_
*s*_1_	Non	Non	Non	*q*_1,4_ = 0.007	*q*_1,5_ = 0.007	Non	Non	Non
*s*_2_	Non	Non	Non	*q*_2,4_ = 0.013	Non	Non	Non	Non
*s*_3_	Non	Non	*q*_3,3_ = 0.013	Non	*q*_3,5_ = 0.027	*q*_3,6_ = 0.013	Non	Non
*s*_4_	*q*_4,1_ = 0.007	*q*_4,2_ = 0.013	*q*_4,3_ = 0.027	*q*_4,4_ = 0.267	*q*_4,5_ = 0.087	*q*_4,6_ = 0.007	*q*_4,7_ = 0.007	*q*_4,8_ = 0.007
*s*_5_	*q*_5,1_ = 0.007	Non	Non	*q*_5,4_ = 0.087	*q*_5,5_ = 0.233	*q*_5,6_ = 0.040	*q*_5,7_ = 0.020	*q*_5,8_ = 0.007
*s*_6_	Non	Non	*q*_6,3_ = 0.013	*q*_6,4_ = 0.007	*q*_6,5_ = 0.047	Non	Non	Non
*s*_7_	Non	Non	Non	*q*_7,4_ = 0.007	*q*_7,5_ = 0.020	Non	Non	Non
*s*_8_	Non	Non	Non	*q*_8,4_ = 0.007	*q*_8,5_ = 0.007	Non	Non	Non

## Data Availability

Not applicable.
